# Targeting RGS4 Ablates Glioblastoma Proliferation

**DOI:** 10.3390/ijms21093300

**Published:** 2020-05-07

**Authors:** Maheedhara R. Guda, Kiran K. Velpula, Swapna Asuthkar, Charlie P. Cain, Andrew J. Tsung

**Affiliations:** 1 Department of Cancer Biology and Pharmacology, University of Illinois College of Medicine at Peoria, Peoria, IL 61605, USA; gmreddy@uic.edu (M.R.G.); velpula@uic.edu (K.K.V.); asuthkar@uic.edu (S.A.); cpcain2@uic.edu (C.P.C.); 2 Department of Neurosurgery, University of Illinois College of Medicine at Peoria, Peoria, IL 61605, USA; 3 Department of Pediatrics, University of Illinois College of Medicine at Peoria, Peoria, IL 61605, USA; 4 Illinois Neurological Institute, Peoria, IL 61605, USA

**Keywords:** RGS4, MMP2, glioma stem cells, apoptosis

## Abstract

Glioblastoma (GBM) is the most common type of adult primary brain tumor with a median survival rate of less than 15 months, regardless of the current standard of care. Cellular heterogeneity, self-renewal ability and tumorigenic glioma cancer stem cell (GSC) populations contribute to the difficulty in treating GBM. G-protein-coupled receptors (GPCRs) are the largest group of membrane proteins and mediate many cellular responses. Regulators of G-protein signaling 4 (RGS4) are negative regulators of G-protein signaling, and elevated levels of RGS4 are reportedly linked with several human diseases, including cancer. This study investigates the effect of silencing RGS4, resulting in inhibition of GSC growth, invasion and migration. Data obtained from The Cancer Genome Atlas (TCGA) demonstrated poor patient survival with high expression of RGS4. Immunohistochemistry and immunoblot analysis conducted on GBM patient biopsy specimens demonstrated increased RGS4 expression correlative with the TCGA data. RNA sequencing confirmed a significant decrease in the expression of markers involved in GSC invasion and migration, particularly matrix metalloproteinase-2 (MMP2) in knockout of RGS4 using CRISPR plasmid (ko-RGS4)-treated samples compared to parental controls. Gelatin zymography confirmed the reduced activity of MMP2 in ko-RGS4-treated samples. Silencing RGS4 further reduced the invasive and migratory abilities and induction of apoptosis of GSCs as evidenced by Matrigel plug assay, wound healing assay and human apoptosis array. Collectively, our results showed that the silencing of RGS4 plays an important role in regulating multiple cellular functions, and is an important therapeutic target in GBM.

## 1. Introduction

Glioblastoma multiforme (GBM) is a highly malignant tumor derived from glial cells and is the most common adult brain tumor. The median survival rates, despite the advances in standard of care, such as total surgical resection, chemotherapy and radiation therapy, still remain approximately 15 months [1,2]. This poor prognosis is due to a number of factors, including tumor recurrence and glioma stem cells (GSC) [3]. These GSCs are the subpopulation of cells present in the tumors, capable of self-renewal, differentiation and tumorigenicity [4]. Since GSCs contribute to GBM initiation, progression and recurrence, manipulating GSCs represents a strategy for novel therapies against GBM [5].

G-protein-coupled receptors (GPCRs) are well known and ubiquitous targets [6,7]. Forming the largest family of membrane receptors, they are involved in a wide variety of physiological functions, including regulation of cellular motility, growth, differentiation and gene expression [8,9,10,11,12,13,14,15,16,17]. GPCRs are reported to play crucial roles in the malignant transformation of human cancers [18]. The downstream signaling of G-protein-coupled receptors is modulated by regulator of G-protein signaling (RGS) proteins that catalyze the dephosphorylation of guanosine triphosphate into guanosine diphosphate. Following cleavage of the third phosphate molecule and subsequent release of energy, regulator of G-protein signaling 4 (RGS4) locks the G-protein in its inactive guanosine diphosphate (GDP) state [19,20]. Here, we hypothesize that glioblastoma (GBM) tumors rely on GPCR regulation, making RGS4 an excellent candidate as a tumor-promoting gene.

RGS4′s implication in cancer is not well documented, other than as a novel regulator in malignant melanoma [21]. RGS4 has recently been studied as a tumor promoter in glioma cells by acting positively on tumor cell motility, and is reported to be a potent driver of human glioma cell invasiveness [22]. In lieu of the aforementioned findings, we hypothesize that RGS4 propagates glioma tumor cell migration and invasion. These effects are accomplished by working in concert with the upregulation of matrix metalloproteinase-2 (MMP2), which is implicated in tumor-associated tissue remodeling via disruption of the extracellular matrix [23,24,25,26], ultimately promoting glioma cell invasion and apoptosis evasion [27,28,29,30,31,32]. Several investigations have indicated that the *MMP2* gene is a significant marker related to glioma formation. In addition, it has been reported that *MMP2* overexpression is associated with brain tumor malignancy and metastasis. Contrary to this, MMP2 absence in GBM-bearing mice showed better survival, further implicating its role in GBM proliferation [33,34,35].

In the present study, we hypothesized that RGS4 overexpression in GSCs promotes GBM invasion, migration and apoptosis evasion via simultaneous expression and production of matrix metalloproteinases, and silencing of RGS4 via CRISPR plasmids knockout-RGS4 (ko-RGS4) reduced GSC invasion and migration. RGS4 expression via immunohistochemistry is comparable to the data obtained from The Cancer Genome Atlas (TCGA) data. RNA sequencing analysis performed using ko-RGS4 treatment showed a significant reduction in the expression levels of MMP2, one of the candidate molecules reported to be downregulated, when compared to the untreated parental controls. Silencing RGS4 significantly reduced the expression and activity of MMP2 as determined by immunoblot and gelatin zymography. Further functional analysis using silencing plasmids of RGS4 showed reductions in invasive potential, migratory capacity and induction of apoptosis. These results show that RGS4–MMP2 signaling may be involved in promoting invasion, migration and propagation of GSCs. Since the proproliferative mechanism is active in GSCs, this may represent a therapeutic target of interest.

## 2. Results

### 2.1. RGS4 Is Overexpressed in Glioblastoma

**Figure 1:**
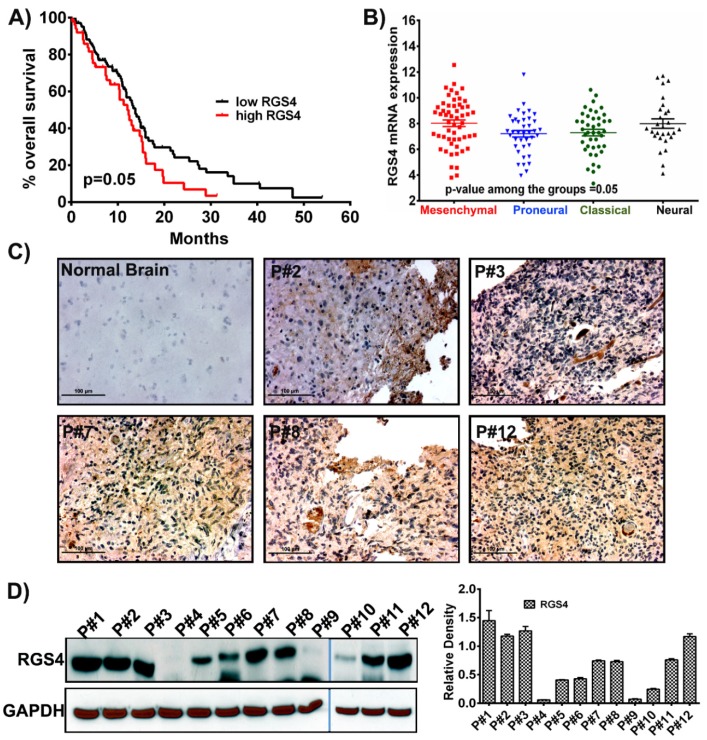
RGS4 expression in glioblastoma samples. (**A**) Kaplan–Meier curve plotted from the data obtained from The Cancer Genome Atlas (TCGA) shows that increased regulators of G-protein signaling 4 (RGS4) expression corresponds to decreased survival (*n* = 172). *p*-value is calculated by log rank test (*p* = 0.05). (**B**) The mRNA expression levels of RGS4 plotted in different subtypes of glioblastoma (*n* = 163; mesenchymal = 42; proneural = 55; classical = 27; neural; = 39). The error bars are plotted based on the standard error of mean (SEM). (**C**) Immunohistochemical staining for RGS4 with anti-RGS4 antibody (brown, diaminobenzidine; light blue, nuclear counter stain with 4′,6-diamidino-2-phenylindole (DAPI); P = glioblastoma specimen) showed positive staining in the glioblastoma (GBM) patient specimens. Negative staining is observed in the normal brain sample specimen (Bar = 100 µM). (**D**) Immunoblot analysis of RGS4 expression in different patient samples (human GBM (hGBM) patient samples = 12). GAPDH (Glyceraldehyde 3-phosphate dehydrogenase) is used as a loading control. The density levels were quantified and represented as a bar graph.

Recent studies conducted on RGS4 have illustrated its role in tumor development and in diverse pathologies, including CNS diseases, cardiovascular disease and diabetes [21,22,36,37]. However, very little information is reported regarding its role in glioblastoma. To better understand the effect and role of RGS4 in glioblastoma prognosis, we opted for a data mining approach. Using the data obtained from the TCGA GBM cohort and glioblastoma biodiscovery portal, we plotted the overall survival by dichotomizing all the patients into two groups, based on the median of *RGS4* gene expression, both low and high. Kaplan–Meier survival analysis showed the significant trend that patients with low RGS4 had higher survival when compared with the patients with high RGS4 (*p* = 0.05) (Figure 1A). Since it is reported that the distinct molecular subtypes of GBM may show a difference in prognosis and responses to treatments, we next examined the expression profile of RGS4 among the four subtypes (mesenchymal, classical, proneural and neural). Group comparison demonstrated that RGS4 expression was highest in the mesenchymal subtype. The classical, proneural and neural subtypes showed comparable RGS4 expression between the subtypes (*p* = 0.05) (Figure 1B). To investigate if RGS4 could be a potential predictor for GBM tumor growth and progression, we performed immunohistochemical analysis on five human glioblastoma (hGBM) patient biopsy tissues, and the positive staining results revealed significantly increased RGS4 expression (Figure 1C). Furthermore, to confirm a correlation between RGS4 staining and protein expression levels, several patients from our own cohort were subjected to immunoblot analysis. Our results showed that RGS4 expression levels were high; however, there was some variability as P# 4, 9 and 10 showed relatively low expression in this patient cohort. The immunohistochemical staining correlated with the immunoblot analysis (Figure 1D). These aforementioned results suggest that RGS4 is highly expressed in mesenchymal subtyped glioblastoma, and understanding its role in GBM will be novel.

### 2.2. RNA Sequencing (RNA-Seq) to Identify MMP2 as a Downstream Effector Gene of RGS4 Signaling

**Figure 2:**
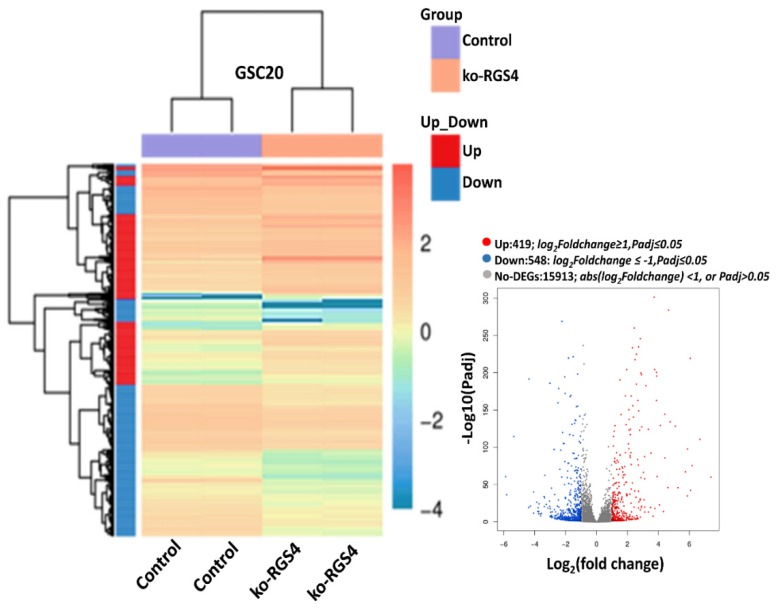
RNA sequencing analysis. Hierarchical clustering RNA sequencing (RNA-seq) data from untreated control and ko-RGS4-treated glioma cancer stem cell (GSC)20 cells. The gradient legend at the top right of the graph represents the FPKM (fragments per kilobase of exon model per million reads mapped) value that has been logarithmically converted. Each column represents a sample, each row represents a gene, different colors represent different expression levels—red for high expression and blue for low expression. Inlet: Volcano plot of differentially expressed genes (DEGs) (red = highly expressed; blue = highly downregulated; grey = unchanged).

**Figure 3:**
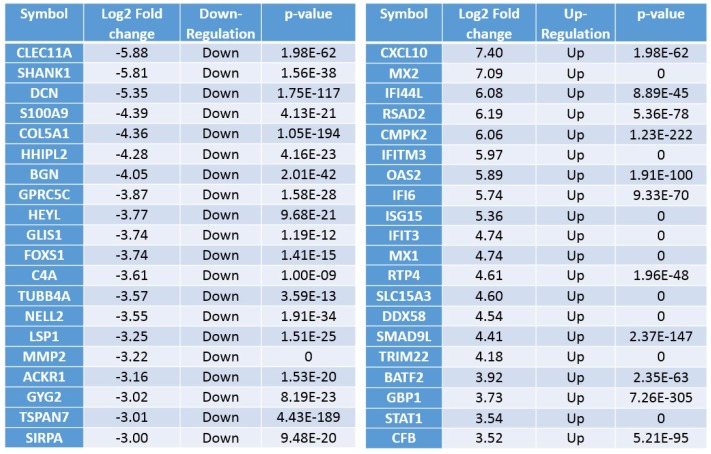
Differentially expressed genes. Top upregulated and downregulated genes when silencing RGS4 in GSC20 cells.

To identify potential downstream signaling targets of RGS4, knockout-RGS4 (ko-RGS4) lines were generated in GSC20 cells using CRISPR/Cas9 (CRISPR associated protein 9) constructs from commercial supplier GeneCopoea. The green fluorescent protein (GFP)-positive-transfected cells were amplified using neomycin selection (1 mg/mL) for about three weeks. The neomycin-resistant single cell clones/colonies were then isolated using cloning cylinders and expanded further. The expanded clones were then validated by microscopic evaluation of the GFP reporter and by performing immunoblot analysis. Out of six clones, clones 2 and 3 lacking RGS4 expression were identified and clone 2 was used in all of the experiments performed in this study (Figure S1). To delineate the effect of global RSG4 knockout, we performed transcriptome RNA sequencing analysis. Bioinformatic analysis confirmed 967 differentially expressed genes (DEGs) comprising 548 downregulated and 419 upregulated genes in the ko-RGS4-treated samples when compared to the untreated GSC20 cells. We next performed hierarchical clustering of DEGs amongst the groups, which revealed tight clustering, suggesting a close correlation of the DEGs between groups, as well as high reproducibility in biological replicates (Figure 2). Several members of the matrix assembly such as decorin (DCN); collagen, type V, alpha 1 (COL5A1); biglycan (BGN) and matrix metalloproteinase-2 (MMP2) were significantly downregulated in the GSC treated with ko-RGS4. Differential expression analysis of genes in the untreated parental controls were compared with ko-RGS4 treatments and the results were represented using a volcano plot (Figure 2 inset). Red and blue points mark the genes with significantly increased or decreased expression, respectively, in GSC20 control and ko-RGS4. Interestingly, most of the interferon family or interferon-regulating genes such as *CXCL10, MX2, MX1, RSAD2, IFI445* and *IFITM3* were markedly upregulated in the ko-RGS4-treated GSC20 cells when compared to control (Figure 3).

### 2.3. RGS4 Knockout Reduces the Expression of MMP2

**Figure 4:**
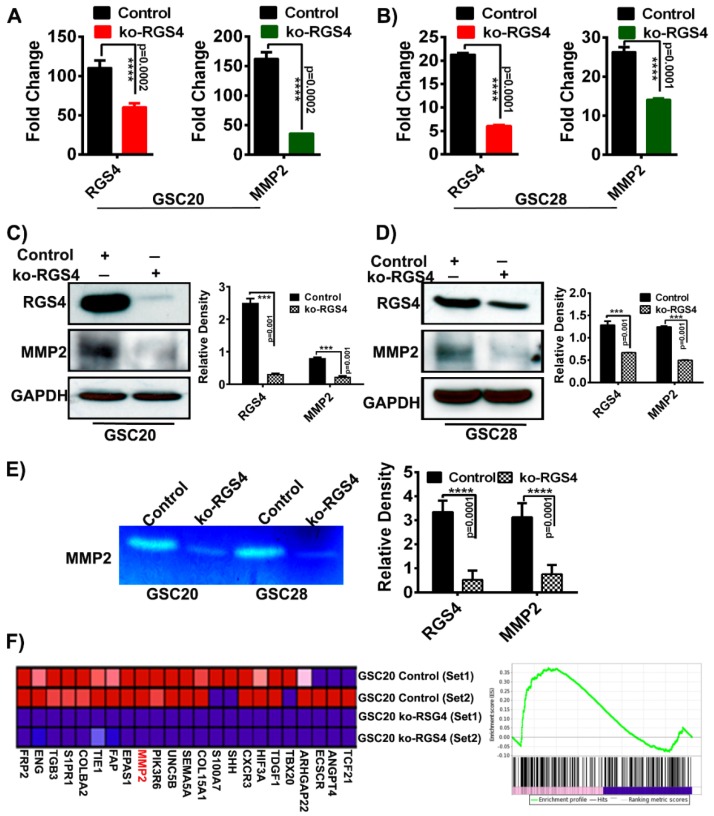
RGS4 modulate matrix metalloproteinase-2 (MMP2) expression. RT-PCR and immunoblot analysis of RGS4 and MMP2 in GSC20 and GSC28 cells treated with knockout-RGS4 (ko-RGS4) and untreated control cells (**A**–**D**) (*n* = 3) (**** *p* = 0.0002; **** *p* = 0.0001; *** *p* = 0.001). GAPDH was used as a loading control. The density levels were quantified and represented as a bar graph. (**E**) MMP2 activity was evaluated by gelatin zymography by using conditioned media from control and ko-RGS4-transfected GSC20 and GSC28 cells collected 48 h after transfection. Bar graph represents the densitometry analysis of MMP2 activity. (**F**) Gene set enrichment analysis (GSEA) using gene set involved in maintaining GSC angiogenesis after ko-RGS4 treatment. The green line demonstrate the enrichment profile. The error bars are plotted based on the SEM (standard error of mean).

It has been reported previously most GSCs express high levels of the gelatinase MMP2 that is synthesized as a proenzyme and is subsequently activated upon cleavage. The overexpression of MMP2 in GSCs is also known to promote GBM invasion [38]. We observed that the expression of MMP2 was reduced by –3.22 log2 fold with a *p*-value of 0 upon ko-RGS4 treatment (Figure 3). To confirm and validate the data obtained from our RNA-seq analysis, we performed real-time PCR analysis on GSC20 and GSC28 cells using untreated control and ko-RGS4 treatments. We observed a significant reduction in the expression level of both RGS4 and MMP2 and this was in agreement with the RNA-seq results (Figure 4A,B). Next, we performed immunoblot analysis on the aforementioned treatments and observed correlative results with the RT-PCR data (Figure 4C,D). As we observed reduced levels of MMP2 both in the RT-PCR and immunoblot analysis, we performed gelatin zymography, a functional assay used to detect gelatinases in culture supernatants of both the control and ko-RGS4-treated GSC20 and GSC28 cells. Analysis of the presence of gelatinases by zymography revealed high levels of MMP2 in the supernatants of both control cell cultures of GSC20 and GSC28, whereas ko-RGS4 treatment demonstrated a significant reduction of MMP2 activity (Figure 4E). Next, we reported changes in the levels of several genes involved in the maintenance of GSC angiogenesis, including MMP2 following ko-RGS4 treatment as a heat map (Figure 4F). The gene set enrichment analysis (GSEA) score plot further shows a significant and positive correlation with our immunoblot and zymography data.

### 2.4. RGS4 Knockout Reduces GSC Migration, Invasion and Induces Apoptosis in GSC

**Figure 5:**
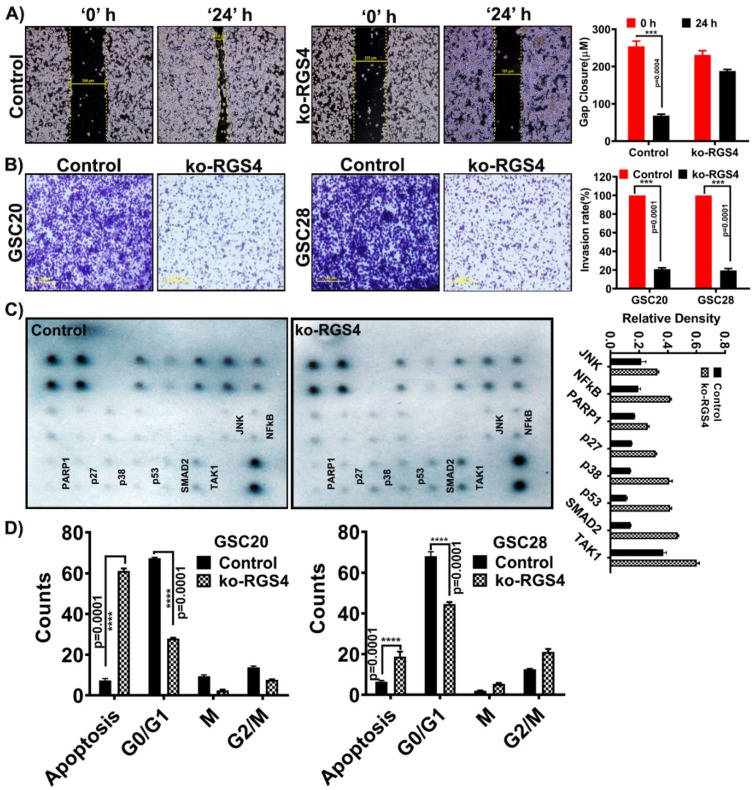
Functional analysis of targeting RGS4 in GSC. (**A**) GSC20 cells were cultured on Poly-D-Lysine-coated six-well plate and treated with ko-RGS4. After 48 h of transfection, a straight scratch was made in individual wells. This point was considered 0 h, and the width of wound was photographed under a microscope. After 24 h, cells were checked for wound healing and again photographed under a microscope. Wound closure distances were used to plot the bar graph (*p* = 0.001, *n* = 3) (*** *p* = 0.004). Yellow bar explains the percentage of wound contraction/repair (Bar = 100 µM). (**B**) Using Matrigel plug invasion assay, ko-RGS4-treated GSC20/GSC28 cells and untreated control cells were plated in a six-well plate. After 24 h, cells penetrating the membrane were fixed and stained with 0.1% crystal violet. The pictograms were captured using an Olympus IX71 microscope. The percent invasion was calculated by taking the untreated control cell invasion as 100% and the treatments were compared against the controls. The data are presented as a bar graph (*p* = 0.001, *n* = 3) (*** *p* = 0.001) (Bar = 100 µM). (**C**) Using a human apoptosis signaling pathway array (Ray Biotech AAH-APOSIG), we recorded increased expression levels of apoptosis markers such as PARP1, p27, p53, SMAD and TAK1 and other markers in the ko-RGS4-treated cells when compared to the untreated control cells. The band densities were plotted in a bar graph (*n* = 2). (**D**) Cell cycle analysis using flow cytometry confirmed increased apoptosis upon ko-RGS4 treatment. Graphical representation of the percentage of cells in each stage of the cell cycle was represented in a bar graph (*n* = 3) (**** *p* = 0.0001). The error bars are plotted based on the SEM (standard error of mean).

To study if ko-RGS4 play a role in modulating GSC migration, we performed a wound healing scratch assay. Both GSC20 and GSC28 cells were triturated into a single cell suspension and plated on Poly-D-Lysine-coated plates. A scratch was made using a 200 µL pipette tip, and the wound closure in untreated control cells and ko-RGS4-treated cells was measured between 0 and 24 h. Treatment with ko-RGS4 inhibited wound healing by more than 70% when compared to control GSC (Figure 5A). 

Next, to test whether silencing of RGS4 modulates cell invasion, we performed an in vitro cell Matrigel invasion assay. The invasion capacities of both ko-RGS4-treated GSC20 and GSC28 cells were reduced significantly when compared to the untreated parent controls (Figure 5B). We next used human apoptosis signaling pathway array C1 (Ray Biotech, cat# AAH-APOSIG-1-4) to study the effect of silencing ko-RGS4 in GSC20 cells. Upon treatment, we recorded increased expression levels of cleaved PARP1, p27, p38, p53, SMAD2, TAK1 and NFκB, indicative of the induction of apoptosis (Figure 5C). Fluorescence activated cell sorting (FACS) analysis further showed increased apoptosis with reduced proliferation in the ko-RGS4-treated samples when compared to untreated controls of GSC20 and GSC28 cells (Figure 5D).

## 3. Discussion

The regulator of G-protein signaling proteins of the R4 family play a regulatory role in the central nervous system and in tumorigenesis. Antagonizing its overexpression in GBM may be an attractive strategy for oncotherapy. A recent body of literature suggests the involvement of RGS4 in breast [39] and non-small cell lung (NSCL) cancers [40]; however, the physiological function of RGS4 in GBM and specifically in GSCs is not known, with little published data to date [41]. Our studies demonstrate that RGS4 is highly expressed in clinical specimens of glioblastoma substantiated by the TCGA data. Silencing RGS4 antagonizes GSC invasion and migration, and identifies a novel and potentially important role for inducing apoptotic cell death.

A small population of neural stem-like cells within the tumor, termed GSCs, are known to recapitulate glioma and its growth characteristics. Study of GSCs is important as they are resistant to traditional treatments and have the ability to self-renew, initiate tumor growth and recur [42,43]. Molecular classification of GBM identified four subtypes: classical, proneural, neural and mesenchymal, with the mesenchymal subtype displaying a more aggressive phenotype and pronounced radio/chemoresistance. Recent literature showed that the proneural tumors tend to shift towards a mesenchymal subtype upon recurrence, induced by radiation/chemotherapies [44,45]. The TCGA analysis performed utilizing mRNA of RGS4 demonstrated increased expression in the mesenchymal subtype when compared with different subgroups. We confirmed these findings by checking the expression levels of RGS4 in both classical (GSC 6-27) and mesenchymal (GSC20 and GSC28) stem cells. Interestingly, RSG4 is not detected in the GSC 6-27 cells, while abundant expression was recorded in the mesenchymal subtyped GSC (data not shown). To generate a comprehensive analysis of targeting RGS4 in mesenchymal GSC and to recognize the critical downstream effectors of RGS4, RNA sequencing analysis was performed. Gene expression profiling and hierarchal clustering of the RNA-seq identified the regulation of key targets involved with GSC migration. Silencing RGS4 controlled the expression of SHANK (SH3 and multiple ankyrin repeat domains) proteins, critical regulators of G-protein signaling that are known to antagonize integrin activation disrupting G-protein interactions along the Rap1-RIAM (Rap1 interacting adaptor molecule)-talin axis in cancer cells and neurons [46]. Targeting RGS4 likewise diminished the expression of decorin, an extracellular matrix (ECM) protein engaged in the cell growth, differentiation, proliferation, adhesion and metastasis of various cancers [47]. Matrix metalloproteinases (MMPs) are the essential agents responsible for ECM degradation, and their abnormal expression is linked to the progression of many cancers, including glioblastoma. Since MMP2 is known for its role in ECM degradation, we selected MMP2 as a candidate that could be studied in the context of RGS4 silencing. Here, we found that RGS4 silencing decreased the expression, secretion and activity of MMP2, a key MMP associated with tumor dissemination and invasiveness. Therefore, RGS4 may affect the expression of MMPs through the GPCR signaling pathway.

Our results showed that silencing RGS4 demonstrated decreased migratory and invasive abilities of both GSC20 and GSC28. We furthered our understanding by utilizing the apoptosis array and recorded the expression of several antiapoptotic molecules involved in the induction of apoptotic effects in GBM. p53 is reported to regulate the transcription of genes associated with cell cycle invasion and apoptosis. Induction of apoptosis and inhibition of cell proliferation are correlated with RGS4 silencing, leading to induction of the subG0 phase of the cell cycle. Silencing of RGS4 may also induce oxidative stress, leading to the activation of JNK, resulting in the induction of apoptosis. Further RGS4 suppression increased PARP1 and p27 signaling, implicating RGS4 in GSC cell cycle regulation and DNA repair leading to apoptosis.

In conclusion, these results show that silencing RGS4 alters cell growth, migration and invasion of GSCs. This study additionally provides insights into the functional relationship between RGS4 depletion leading to MMP2 degradation. We also infer that RGS4 regulate the expression, secretion and activity of MMP2 in GSCs. In both GSC20 and GSC28 cells, the expression of p27, JNK, p53 and PARP1 upon silencing of RGS4 demonstrate the anticancer effect, orchestrated by cell cycle alterations and the activation of apoptosis. These findings establish RGS4 as the promoter of invasive behavior of GSCs, and its silencing mechanisms can be used to curtail GBM growth and progression.

## 4. Materials and Methods

### 4.1. Ethics Statement

Human GBM surgical biopsy specimens were obtained from OSF Saint Francis Medical Center, Peoria, IL, USA and processed in accordance with the University of Illinois College of Medicine at Peoria (UICOMP) Institutional Review Board-approved protocol (protocol# 85193).

### 4.2. Cell Culture and Reagents

Two different GBM GSC cell lines, GSC20 and GSC28, were used in this study. These established and authenticated cell lines as previously reported, were obtained from Dr. Bhat’s Laboratory, MD Anderson Cancer Center, Houston, Texas, USA and were grown in DMEM/F12 supplemented with B27 (Life Technologies, Carlsbad, CA, USA), EGF (20 ng/mL) (Millipore, Sigma, St. Louis, MO, USA), bFGF (20 ng/mL) (Millipore, Sigma) and 1% penicillin/streptomycin (Life Technologies) [44,48,49]. RGS4 CRISPR all-in-one plasmids were obtained from Genecopoea (HCP257325-CG04). Anti-RGS4, anti-MMP2 and anti-GAPDH antibodies were obtained from Santa Cruz (Santa Cruz, CA, USA).

### 4.3. Generation of RGS4 All-In-One CRISPR Plasmids and Construction of RGS4 Knockouts

The all-in-one plasmids intended for a complete knockout of the RGS4 were purchased from Genecopoea. Briefly, the sgRNAs to RGS4 were designed as close to the initiator ATG as possible, and the combination of expression of both Cas9 and sgRNA from each plasmid created a double-strand break (DSB) at the target site, which informed that the sgRNA was functional. This DSB was repaired using nonhomologous end joining (NHEJ), resulting in deletions or small insertions in the protein-coding region of the *RGS4* gene. These lesions caused a frameshift mutation that led to the knockout of the *RGS4* gene. These frameshift mutations occurred on both (or all in polyploid strains) alleles of the gene, resulting in a loss-of-function mutation. The all-in-one expression plasmids targeting RGS4 were transformed into DH5α competent cells and selected on LB agar plates containing 100 mg/mL ampicillin. The transformed plasmids were extracted using a Qiagen maxi prep kit (Valencia, CA, USA). The GSC20 cells were transiently transfected with RGS4 knockout plasmids using Lipofectamine 3000 (ThermoFisher Scientific, Waltham, MA, USA) in a 6-well culture plate. After 72 h of transfection, GSC 20 cells were selected using 1 mg/mL neomycin. Around six neomycin-resistant single cell clones/colonies with the highest GFP expression were obtained and isolated using cloning cylinders (Millipore, Sigma) and expanded. These clones were further validated by microscopic evaluation of GFP expression and by the immunoblot approach. Out of six clones, clones 2 and 3 showed a complete knockout of RGS4 and clone 2 was selected for this study.

### 4.4. TCGA Cohort Analysis

The mRNA expression and clinical data from 172 cases were obtained from the glioblastoma cohort using The Cancer Genome Atlas (TCGA) data portal (http://cancergenome.nih.gov).

### 4.5. Immunohistochemical Analyses of Human Glioblastoma (hGBM) Patient Specimens

The hGBM surgical biopsy specimens were obtained from Saint Francis Medical Center as per the UICOMP-IRB approved protocol 85193 (Peoria, IL, USA) and processed in accordance with the UICOMP Institutional Review Board-approved protocols. Representative GBM sections of P# 2, P# 3, P# 7, P# 8 and P# 12 were used for immunohistochemistry analysis. For RGS4 staining, we used mouse antibody specific for RGS4 at 1:500 dilution. The sections were deparaffinized, rehydrated and blocked in 10% goat serum for an hour. Sections were incubated in the primary antibody solution overnight at 4 °C in a humidified chamber and then washed in 1 × PBS (phosphate buffered saline), incubated with the appropriate secondary antibody for 1 h, visualized and analyzed using a confocal microscope. All immunostained sections, when stained with DAB (3,3’-Diaminobenzidine), were counterstained with hematoxylin. Negative controls were maintained without primary antibody by using IgG antibody. The sections were blind reviewed by a neuropathologist.

### 4.6. Immunoblot Analysis

Total protein extracts from various treatments were resolved by 12% SDS-PAGE and transferred to nitrocellulose membrane, and were blocked in Tris HCl, pH 7.5, 500 mM NaCl and 0.1% Tween 20 containing 5% nonfat milk. The blots were incubated with anti-RGS4 (1:500, Santa Cruz), MMP2 (1:500, Santa Cruz) and GAPDH (1:1000, Santa Cruz) primary antibodies, and subsequently incubated with a 1:2000 dilution of species-specific, horseradish peroxidase (HRP)-conjugated secondary antibody. Imaging and data analysis were carried out using chemiluminescence ECL (Hercules, CA, USA) Western blotting detection reagents on Hyperfilm MP autoradiography film. GAPDH antibody was used to verify equal loading of proteins in all lanes.

### 4.7. RNA-seq Analysis of Knockout of RGS4 (ko-RGS4)

Total RNA samples isolated from the control and ko-RGS4-treated GSC20 were measured using an Agilent 2100 Bioanalyzer (Agilent RNA 6000 Nano Kit) (Santa Clara, CA, USA). The total RNA was then proceeded to cDNA preparation and the obtained single-strand DNA then sequenced on the BGISEQ-500 platform. The mRNA values obtained are presented as a heat map.

### 4.8. Bioinformatic Analysis

The clean quality reads with adaptors were assembled into unigenes, followed by unigene functional annotation and SSR (simple sequence repeats) detection. Finally, the DEGs (differentially expressed genes) between control and treated samples were identified and then followed by clustering analysis and functional annotations. Fold change ≥ 2.00 and adjusted *p*-value ≤ 0.05 were considered for the annotations.

### 4.9. Gene Set Enrichment Analysis (GSEA)

We used gene set enrichment analysis (GSEA, v3.0, Broad Institute) to determine whether GSC control and ko-RGS4-treated cells showed statistically significant differences. All gene sets used for analysis were downloaded from the Broad Institute Molecular Signature Database (mSigDB v6.2, software.broadinstitute.org/gsea/msigdb). 

### 4.10. RT-PCR Analysis

Total RNA was isolated from both GSC20 and GSC28 cells transfected with a CRISPR plasmid of RGS4 (ko-RGS4). RT-PCR was conducted from the control and transfected mRNA using the SYBR green method. The primers used in the study were RGS4 sense 5′-ACATCGGCTAGGTTTCCTGC-3′ and antisense 5′-GTTGTGGGAAGAATTGTGTTCAC-3′; MMP2 sense 5′-GGCCCTGTCACTCCTGAGAT-3′ and antisense 5′-GGCATCCAGGTTATCGGG GA-3′; GAPDH sense 5′-AATCCCATCACCATCTTCCA-3′ and antisense 5′-TGGACTCCACGACGT ACTCA-3′.

### 4.11. Gelatin Zymography for Assaying Matrix Metalloproteinase-2

We used gelatin zymography to assess the enzymatic activity of MMP2. The culture supernatants from both GSC20 and GSC28 untreated control cells and ko-RGS4-transfected cells were collected after 48 h. The supernatants were centrifuged at 3000 rpm for 10 min. The proteins in the samples were separated by SDS-PAGE. After electrophoresis, the SDS-PAGE gel was proceeded further with the buffers and staining reagents according to Abcam’s protocol (https://www.abcam.com/protocols/gelatinzymography protocol). The intensity of the bands from both the control and the treated samples were analyzed by ImageJ Software (National Institutes of Health, Bethesda, MD, USA).

### 4.12. Wound Healing and Matrigel Invasion Assays

To study GSC motility, we performed the wound healing assay using GSC20 and GSC28 cells. We used Corning™ BioCoat™ Poly-D-Lysine 6-well plates (cat# 08-774-123) for this assay. Around 1 × 10^6^ GSC20 and GSC28 cells were plated on the Poly-D-Lysine plate 12 h before the transfection. After 48 h, the monolayers of both cells were scratched using a sterile 200 μL pipette tip with a constant width. Both the control and ko-RGS4-transfected cells were photographed at 0 and 24 h. The distances were measured using the following formula:

Distance migrated = (distance at 0 h − distance at 24 h)/distance at 0 h × 100% [50].

Matrigel invasion assay was used to assess GSC invasive potential. DMEM/F12 medium with supplements was added to the lower chamber to act as a chemoattractant. Both GSC20 and GSC28 control cells and ko-RGS4-transfected cells were seeded at a density of 2 × 10^5^ cells per well onto the 8 μm pore size upper inserts and then incubated at 37 °C. After 24 h, the noninvasive cells were removed from the upper surface of the separating membrane by gentle scrubbing with a cotton swab, and the invading cells were fixed in 100% methanol and stained with HEMA-3 stain. The pictograms were captured using an Olympus IX71 microscope (Center Valley, PA, USA).

### 4.13. Human Apoptosis Signaling Pathway Array and Flow Cytometric Analysis

Around 300 µg of GSC20 control and ko-RGS4-treated total cell lysates were subjected to human apoptosis signaling pathway array C1 (Ray Biotech, cat# AAH-APOSIG-1-4) by following the manufacturer’s instructions. Changes in cell cycle upon ko-RGS4 were analyzed using flow cytometry analysis. Cell cycle progression and DNA content were monitored by staining with propidium iodide and were carried out with a fluorescence-activated cell sorter (FACS Aria; Becton Dickinson, San Jose, CA, USA). The percentage of cells within subG0, G1, S, G2 and M phases was determined using CellQuest software (BD Biosciences, San Jose, CA, USA). Approximately 10,000 events were counted for each analysis, and experiments were performed in triplicate for each group (*n* = 3).

### 4.14. Statistical Analysis

The results shown are represented as mean ± SD. GraphPad 8.0 was used to perform Student’s t-tests and ANOVA to evaluate the differences between the control and treated groups. For multiple comparisons within TCGA, we used Student’s t-test to calculate the expressional differences. All *p*-values were considered statistically significant with a value < 0.05. ImageJ Software was used for all the densitometry analyses.

## Supplementary Materials

Supplementary materials can be found at https://www.mdpi.com/1422-0067/21/9/3300/s1. Figure S1. Clonal selection of RGS4 knockouts.

## References

[1] Stupp R., Mason W.P., van den Bent M.J., Weller M., Fisher B., Taphoorn M.J., Belanger K., Brandes A.A., Marosi C., Bogdahn U. (2005). Radiotherapy Plus Concomitant and Adjuvant Temozolomide for Glioblastoma. N. Engl. J. Med..

[2] Stupp R., Hegi M.E., Mason W.P., van den Bent M.J., Taphoorn M.J., Janzer R.C., Ludwin S.K., Allgeier A., Fisher B., Belanger K. (2009). Effects of Radiotherapy with Concomitant and Adjuvant Temozolomide Versus Radiotherapy Alone on Survival in Glioblastoma in a Randomised Phase III Study: 5-Year Analysis of the EORTC-NCIC Trial. Lancet Oncol..

[3] Yamashita D., Bernstock J.D., Elsayed G., Sadahiro H., Mohyeldin A., Chagoya G., Ilyas A., Mooney J., Estevez-Ordonez D., Yamaguchi S. (2020). Targeting Glioma-Initiating Cells Via the Tyrosine Metabolic Pathway. J. Neurosurg..

[4] Liebelt B.D., Shingu T., Zhou X., Ren J., Shin S.A., Hu J. (2016). Glioma Stem Cells: Signaling, Microenvironment, and Therapy. Stem Cells Int..

[5] Gimple R.C., Kidwell R.L., Kim L.J.Y., Sun T., Gromovsky A.D., Wu Q., Wolf M., Lv D., Bhargava S., Jiang L. (2019). Glioma Stem Cell Specific Super Enhancer Promotes Polyunsaturated Fatty Acid Synthesis to Support EGFR Signaling. Cancer Discov..

[6] Ramesh M., Soliman M.E. (2015). G-Protein Coupled Receptors (GPCRs): A Comprehensive Computational Perspective. Comb. Chem. High Throughput Screen.

[7] Spiegelberg B.D., Hamm H.E. (2007). Roles of G-Protein-Coupled Receptor Signaling in Cancer Biology and Gene Transcription. Curr. Opin. Genet. Dev..

[8] Hawley J.A., Noakes T.D. (1992). Peak Power Output Predicts Maximal Oxygen Uptake and Performance Time in Trained Cyclists. Eur. J. Appl. Physiol. Occup. Physiol..

[9] Cheng Y.C., Scotting P.J., Hsu L.S., Lin S.J., Shih H.Y., Hsieh F.Y., Wu H.L., Tsao C.L., Shen C.J. (2013). Zebrafish Rgs4 is Essential for Motility and Axonogenesis Mediated by Akt Signaling. Cell Mol. Life Sci..

[10] Yanagida K., Ishii S. (2011). Non-Edg Family LPA Receptors: The Cutting Edge of LPA Research. J. Biochem..

[11] Pierce K.L., Premont R.T., Lefkowitz R.J. (2002). Seven-Transmembrane Receptors. Nat. Rev. Mol. Cell Biol..

[12] Li S., Huang S., Peng S.B. (2005). Overexpression of G Protein-Coupled Receptors in Cancer Cells: Involvement in Tumor Progression. Int. J. Oncol..

[13] Schoneberg T., Schulz A., Biebermann H., Hermsdorf T., Rompler H., Sangkuhl K. (2004). Mutant G-Protein-Coupled Receptors as a Cause of Human Diseases. Pharmacol. Ther..

[14] Cattaneo F., Guerra G., Parisi M., De Marinis M., Tafuri D., Cinelli M., Ammendola R. (2014). Cell-Surface Receptors Transactivation Mediated by G Protein-Coupled Receptors. Int. J. Mol. Sci..

[15] Wang K., Wong Y.H. (2009). G Protein Signaling Controls the Differentiation of Multiple Cell Lineages. Biofactors.

[16] Schwindinger W.F., Robishaw J.D. (2001). Heterotrimeric G-Protein Betagamma-Dimers in Growth and Differentiation. Oncogene.

[17] Ho M.K., Su Y., Yeung W.W., Wong Y.H. (2009). Regulation of Transcription Factors by Heterotrimeric G Proteins. Curr. Mol. Pharmacol..

[18] Choi H.Y., Saha S.K., Kim K., Kim S., Yang G.M., Kim B., Kim J.H., Cho S.G. (2015). G Protein-Coupled Receptors in Stem Cell Maintenance and Somatic Reprogramming to Pluripotent Or Cancer Stem Cells. BMB Rep..

[19] Ghil S., McCoy K.L., Hepler J.R. (2014). Regulator of G Protein Signaling 2 (RGS2) and RGS4 Form Distinct G Protein-Dependent Complexes with Protease Activated-Receptor 1 (PAR1) in Live Cells. PLoS ONE.

[20] Zhang P., Kofron C.M., Mende U. (2015). Heterotrimeric G Protein-Mediated Signaling and its Non-Canonical Regulation in the Heart. Life Sci..

[21] Xue X., Wang L., Meng X., Jiao J., Dang N. (2017). Regulator of G Protein Signaling 4 Inhibits Human Melanoma Cells Proliferation and Invasion through the PI3K/AKT Signaling Pathway. Oncotarget.

[22] Weiler M., Pfenning P.N., Thiepold A.L., Blaes J., Jestaedt L., Gronych J., Dittmann L.M., Berger B., Jugold M., Kosch M. (2013). Suppression of Proinvasive RGS4 by mTOR Inhibition Optimizes Glioma Treatment. Oncogene.

[23] Rao J.S. (2003). Molecular Mechanisms of Glioma Invasiveness: The Role of Proteases. Nat. Rev. Cancer.

[24] VanMeter T.E., Rooprai H.K., Kibble M.M., Fillmore H.L., Broaddus W.C., Pilkington G.J. (2001). The Role of Matrix Metalloproteinase Genes in Glioma Invasion: Co-Dependent and Interactive Proteolysis. J. Neurooncol..

[25] Yong V.W. (2005). Metalloproteinases: Mediators of Pathology and Regeneration in the CNS. Nat. Rev. Neurosci..

[26] Yong V.W., Power C., Forsyth P., Edwards D.R. (2001). Metalloproteinases in Biology and Pathology of the Nervous System. Nat. Rev. Neurosci..

[27] Du R., Petritsch C., Lu K., Liu P., Haller A., Ganss R., Song H., Vandenberg S., Bergers G. (2008). Matrix Metalloproteinase-2 Regulates Vascular Patterning and Growth Affecting Tumor Cell Survival and Invasion in GBM. Neuro Oncol..

[28] Gondi C.S., Dinh D.H., Klopfenstein J.D., Gujrati M., Rao J.S. (2009). MMP-2 Downregulation Mediates Differential Regulation of Cell Death Via ErbB-2 in Glioma Xenografts. Int. J. Oncol..

[29] Mignatti P., Rifkin D.B. (1993). Biology and Biochemistry of Proteinases in Tumor Invasion. Physiol. Rev..

[30] Forsyth P.A., Wong H., Laing T.D., Rewcastle N.B., Morris D.G., Muzik H., Leco K.J., Johnston R.N., Brasher P.M., Sutherland G. (1999). Gelatinase-A (MMP-2), Gelatinase-B (MMP-9) and Membrane Type Matrix Metalloproteinase-1 (MT1-MMP) are Involved in Different Aspects of the Pathophysiology of Malignant Gliomas. Br. J. Cancer.

[31] Kargiotis O., Chetty C., Gondi C.S., Tsung A.J., Dinh D.H., Gujrati M., Lakka S.S., Kyritsis A.P., Rao J.S. (2008). Adenovirus-Mediated Transfer of siRNA Against MMP-2 mRNA Results in Impaired Invasion and Tumor-Induced Angiogenesis, Induces Apoptosis in Vitro and Inhibits Tumor Growth in Vivo in Glioblastoma. Oncogene.

[32] Shastry A.H., Thota B., Arimappamagan A., Santosh V. (2015). P53 Stratification Reveals the Prognostic Utility of Matrix Metalloproteinase-9 Protein Expression in Glioblastoma. Neurol. India.

[33] Wang M., Wang T., Liu S., Yoshida D., Teramoto A. (2003). The Expression of Matrix Metalloproteinase-2 and -9 in Human Gliomas of Different Pathological Grades. Brain Tumor Pathol..

[34] Hu B., Guo P., Fang Q., Tao H.Q., Wang D., Nagane M., Huang H.J., Gunji Y., Nishikawa R., Alitalo K. (2003). Angiopoietin-2 Induces Human Glioma Invasion through the Activation of Matrix Metalloprotease-2. Proc. Natl. Acad. Sci. USA.

[35] Sinceviciute R., Vaitkiene P., Urbanaviciute R., Steponaitis G., Tamasauskas A., Skiriute D. (2018). MMP2 is Associated with Glioma Malignancy and Patient Outcome. Int. J. Clin. Exp. Pathol..

[36] Roman D.L., Traynor J.R. (2011). Regulators of G Protein Signaling (RGS) Proteins as Drug Targets: Modulating G-Protein-Coupled Receptor (GPCR) Signal Transduction. J. Med. Chem..

[37] Ruiz de Azua I., Scarselli M., Rosemond E., Gautam D., Jou W., Gavrilova O., Ebert P.J., Levitt P., Wess J. (2010). RGS4 is a Negative Regulator of Insulin Release from Pancreatic Beta-Cells in Vitro and in Vivo. Proc. Natl. Acad. Sci. USA.

[38] Roos A., Ding Z., Loftus J.C., Tran N.L. (2017). Molecular and Microenvironmental Determinants of Glioma Stem-Like Cell Survival and Invasion. Front. Oncol..

[39] Xie Y., Wolff D.W., Wei T., Wang B., Deng C., Kirui J.K., Jiang H., Qin J., Abel P.W., Tu Y. (2009). Breast Cancer Migration and Invasion Depend on Proteasome Degradation of Regulator of G-Protein Signaling 4. Cancer Res..

[40] He Z., Yu L., Luo S., Li Q., Huang S., An Y. (2019). RGS4 Regulates Proliferation and Apoptosis of NSCLC Cells Via microRNA-16 and Brain-Derived Neurotrophic Factor. Onco Targets Ther..

[41] Heo J.C., Jung T.H., Lee S., Kim H.Y., Choi G., Jung M., Jung D., Lee H.K., Lee J.O., Park J.H. (2016). Effect of Bexarotene on Differentiation of Glioblastoma Multiforme Compared with ATRA. Clin. Exp. Metastasis.

[42] Singh S.K., Clarke I.D., Terasaki M., Bonn V.E., Hawkins C., Squire J., Dirks P.B. (2003). Identification of a Cancer Stem Cell in Human Brain Tumors. Cancer Res..

[43] Singh S.K., Hawkins C., Clarke I.D., Squire J.A., Bayani J., Hide T., Henkelman R.M., Cusimano M.D., Dirks P.B. (2004). Identification of Human Brain Tumour Initiating Cells. Nature.

[44] Bhat K.P.L., Balasubramaniyan V., Vaillant B., Ezhilarasan R., Hummelink K., Hollingsworth F., Wani K., Heathcock L., James J.D., Goodman L.D. (2013). Mesenchymal Differentiation Mediated by NF-kappaB Promotes Radiation Resistance in Glioblastoma. Cancer Cell.

[45] Halliday J., Helmy K., Pattwell S.S., Pitter K.L., LaPlant Q., Ozawa T., Holland E.C. (2014). In Vivo Radiation Response of Proneural Glioma Characterized by Protective p53 Transcriptional Program and Proneural-Mesenchymal Shift. Proc. Natl. Acad. Sci. USA.

[46] Lilja J., Zacharchenko T., Georgiadou M., Jacquemet G., De Franceschi N., Peuhu E., Hamidi H., Pouwels J., Martens V., Nia F.H. (2017). SHANK Proteins Limit Integrin Activation by Directly Interacting with Rap1 and R-Ras. Nat. Cell Biol..

[47] Zhang W., Ge Y., Cheng Q., Zhang Q., Fang L., Zheng J. (2018). Decorin is a Pivotal Effector in the Extracellular Matrix and Tumour Microenvironment. Oncotarget.

[48] Noh H., Zhao Q., Yan J., Kong L.Y., Gabrusiewicz K., Hong S., Xia X., Heimberger A.B., Li S. (2018). Cell Surface Vimentin-Targeted Monoclonal Antibody 86C Increases Sensitivity to Temozolomide in Glioma Stem Cells. Cancer Lett..

[49] Piao Y., Park S.Y., Henry V., Smith B.D., Tiao N., Flynn D.L., de Groot J.F. (2016). Novel MET/TIE2/VEGFR2 Inhibitor Altiratinib Inhibits Tumor Growth and Invasiveness in Bevacizumab-Resistant Glioblastoma Mouse Models. Neuro Oncol..

[50] Zhang L., Sun X., Si J., Li G., Cao L. (2019). Umbelliprenin Isolated from Ferula Sinkiangensis Inhibits Tumor Growth and Migration through the Disturbance of Wnt Signaling Pathway in Gastric Cancer. PLoS ONE.

