# Supplementary material for: Targeting RGS4 Ablates Glioblastoma Proliferation
Source: Int J Mol Sci. 2020 May 7;21(9):3300. doi: 10.3390/ijms21093300 (PMC7247588; doi:10.3390/ijms21093300)
Supplement: Supplementary file 1 [file ijms-21-03300-s001.pdf]

Supplementary Figure 1A:

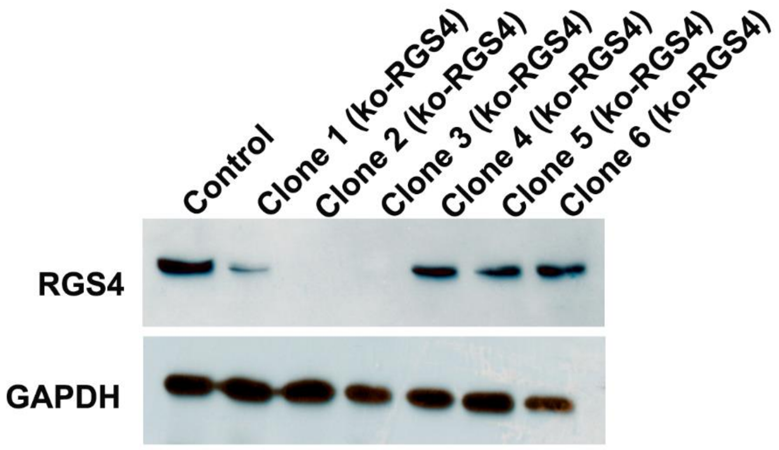

Figure S1. Clonal selection of RGS4 knockouts. GSC20 cells were transfected with all-in-one RSG4 plasmids obtained from Genecopoea. After 72h of transfection, the cells were selected using 1mg/ml of neomycin. Six neomycin selected single cell clones were expanded evaluated by using the immunoblot analysis to assess the knockout of RGS4. Colonies 2 and 3 showed a complete knockout of RGS4 when compared to other colonies. GAPDH was used as a loading control.
